# Serum ferritin levels are associated with frequent consumption of iron- and ascorbate-rich foods among women of childbearing age in Nandi County, Kenya

**DOI:** 10.1017/jns.2022.5

**Published:** 2022-02-08

**Authors:** Patrick Nyamemba Nyakundi, Juliana Kiio, Ann Wambui Munyaka

**Affiliations:** Department of Food, Nutrition and Dietetics, Kenyatta University, Nairobi, Kenya

**Keywords:** Ascorbate-rich foods, Iron-rich foods, Serum ferritin, Women of childbearing age

## Abstract

Information on consumption patterns of iron- and ascorbate-rich foods and their influence on iron status among women of childbearing age (WCA) is scarce in Kenya despite iron deficiency being rampant. The present study investigated consumption patterns of iron- and ascorbate-rich foods on iron status among WCA in Kapsabet Ward, Kenya. The study adopted a cross-sectional analytical design. A sample of 160 respondents was systematically selected proportionately in the eight villages. Consumption patterns of iron- and ascorbate-rich foods were assessed using a modified 7-d Food Frequency Questionnaire. Venous blood (2 ml) was drawn from participants. Serum ferritin and C-reactive proteins were measured by enzyme immunoassay. Consumption patterns of iron- and ascorbate-rich foods were analysed using descriptive statistics. Multivariable regression was conducted to investigate the association between iron- and ascorbate-rich foods consumption and iron status. Confounding variables such as consumption of foods high phytate levels, milk and milk products, recent major blood losses and parasitic infections were controlled for during analysis. The prevalence of iron deficiency among the WCA was 45⋅0 %. Iron-rich foods were rarely (<2 times/week) consumed by the respondents with the majority reporting infrequent consumption: meat (61⋅3 %), sardines (61⋅9 %), oranges (54⋅4 %) and fortified breakfast cereals (94⋅4 %), except for kale and beans. Iron- (iron-fortified porridge, meat, sardines, beans, amaranth and spider plants) and ascorbate- (oranges and mangoes) rich foods positively predicted (AOR = 4⋅851, *P* = 0⋅021) the normal iron status of WCA. WCA should consume above 2 intakes per week of each iron- and ascorbate-rich food for better iron status outcomes.

## Introduction

Twenty-one per cent of women of childbearing age (WCA) in Kenya are iron deficient^(1)^. Iron deficiency is associated with far-reaching consequences in human health, economic development and the social well-being of a population^(2)^. It is recorded to be a key contributor to ‘the global burden of anaemia’^(3)^. Iron deficiency anaemia (IDA) affects maternal and fetal health adversely and is associated with elevated maternal and fetal mortality and morbidity. The affected women usually experience fainting, difficulties in sleeping, breathing difficulties, palpitations and tiredness^(4)^. Also, their risk of developing pre-eclampsia, haemorrhage, perinatal infections, behavioural difficulties and impaired post-partum cognitive functions is increased^(5,6)^. The negative perinatal outcomes suffered may include preterm babies, intrauterine retardation of growth and low birth weight with increased mortality risks^(7)^.

The most prevalent type of anaemia reported is IDA especially among WCA^(8)^. It is said to exist when blood has an inadequate supply of erythrocytes which is mainly caused by the lack of iron that is required for erythropoiesis^(9)^. Since, blood lacks enough erythrocytes, which are primarily engaged in the transportation of oxygen in the body, energy metabolism in cells is impaired leading to tiredness, extreme fatigue, shortness of breath, cold feet and hands, pale skin, chest pain, brittle nails, inflammation of the tongue, headache, light-headedness and poor appetite^(8,10,11)^. In WCA, factors that contribute to IDA include lack of food availability, inadequate dietary diversity, inadequate intake of dietary iron, consumption of poor diets, repeated pregnancies, short intervals between pregnancies and insufficient health systems^(12)^.

Studies conducted in Kenya among pregnant women report their dietary patterns to consist of mashed maize meal^(13,14)^ and sardines^(14)^. Overall, Kiboi *et al.*^(13)^, Smith^(15)^ and Waweru *et al.*^(16)^ reported that women's diets were deficient in iron-rich foods. Two Kenyan studies reported infrequent consumption of iron-rich foods among women^(14,17)^. Similarly, studies from Mumbai in India^(18)^ and both rural and urban areas of Bangladesh^(19)^ have reported a low intake of meat and meat products among WCA. The fact that animal products were generally unpopular among women could compound efforts of supplying sufficient iron to the women as animal products provide haeme iron that has higher bioavailability as compared to non-haeme iron^(20)^. Subsequently, intake of iron supplements and iron has been recorded to be inadequate among expectant women in third-world countries^(15)^.

Since the majority of third-world countries’ women are reported to mainly consume plant-based foods, then strategizing to promote maximal absorption of non-haeme iron is pertinent. Ascorbic acid has been shown to promote the absorption of iron, especially non-haeme iron^(21)^. The ascorbate's enhancing effect can be credited to its ability to transform ferric iron (Fe^3+^) to ferrous iron (Fe^2+^) in the duodenum and stomach to ‘form soluble complexes’ in higher pH of the small intestines to enable its absorption^(22)^. Vegetables and fruits are the richest sources of natural ascorbic acid^(23)^. Fruits rich in ascorbic acid include lemons, oranges, green pepper, papaya, oranges, kiwi, guavas and grapefruit^(24)^. Dark green leafy vegetables such as amaranth, spinach, broccoli, cauliflower, kale and spider plants are rich in natural vitamin C. The seven most popular and readily available fruits in the Kenyan markets include mangoes, oranges, bananas, apples, pawpaws, pineapples and avocados^(25)^. Although most of the fruits in Kenya are seasonal, bananas, oranges and mangoes are readily available most of the time in the year^(26)^. A study conducted in western Kenya established that the favourite fruits among women were mangoes, avocados, oranges, sweet bananas and pawpaws^(27)^. Two Kenyan studies record a low intake of both fruits and vegetables among women way below the recommended level^(27,28)^. Similarly, a study conducted in rural India among women established that fruits were infrequently consumed and below the recommended amounts^(29)^. Many factors could influence the consumption of fruits including the seasonality of their production^(26)^, distribution channels^(29)^ and whether they are local or exotic as well as the price fluctuations.

However, there is a paucity of scientific research carried out in Kenya to understand the consumption pattern of iron- and ascorbate-rich foods. Thus, the present study investigated the consumption pattern of iron- and ascorbate-rich foods and its relationship with iron status among WCA in Nandi County.

## Methodology

An analytical cross-sectional design was adopted to carry out the study among WCA in Nandi County, Kenya. The consumption pattern of iron- and ascorbate-rich foods were the independent variables whereas the iron status of WCA was the dependent variable. Women who were non-pregnant, non-lactating and aged between 15 and 49 years were included in the study. However, respondents who supplemented on iron regularly, recently donated blood (less than 6 months) or were ailing from chronic conditions were excluded from the study.

### Sample size determination and sampling technique

G* Power software version 3.1.9.4 (Universität Düsseldorf, Germany) was used to determine the sample size. The power (1-*β*) and significance (*α*) were set at 0⋅05 and 0⋅95, respectively. The odds ratio for failing to reject the alternative hypothesis ((Pr(*y* = 1|*x* = 1)=H1) was 0⋅65 and OR for rejecting the null hypothesis ((Pr(*y* = 1|*x* = 1)=Ho) was 0⋅35^(30)^. Therefore, the sample size determined was 144 WCA. To account for incomplete questionnaires, 10 % was added to make 160 respondents. Eight villages were determined from Kapsabet Ward by village divisions including Kibabet (9), Township (48), Chemundu (25), Kimonde (12), Kimindamugunya (10), Kiropretmeswo (19), Kimundi (17) and Goitebes (20). Proportionate samples were determined for each village and a systematic sampling technique was used to select participants from respective villages.

### Data collection tools and procedure

A modified food frequency questionnaire (MFFQ) entailing 104 food items was used to collect the consumption patterns of iron- and ascorbate-rich foods. Ten per cent of the sample size^(31)^ selected from Kapsabet Ward was used to conduct a pilot study. The finding of the pilot study informed the modification done on the data collection tool. The test–retest method was used to ensure the reliability of tools. Seven undergraduate nutrition students were recruited and trained on data collection procedures. Also, three qualified phlebotomists were recruited and trained on the blood collection process. Role-plays and demonstrations were used in training until the researcher was satisfied with the data collection competency of the interviewers. During the fieldwork, three teams, consisting of two or three interviewers and one phlebotomist, were dispatched to the assigned villages in Kapsabet Ward. Respondents were systematically selected and informed consent was obtained after which the MFFQ was filled by the interviewers. Blood from the respondents was obtained immediately after the filling of MFFQ.

### Collection of blood sample

The phlebotomist disinfected skin using alcohol swabs. Then, 2 ml of venous blood was collected. 1⋅5 ml of the obtained blood was aliquoted into plain vacutainer tubes. The tubes were coded for identification and then packed in a cooler box (15°C) and sent to Chepsoo Medical Centre for serum separation upon centrifugation. The separated serum was put into vials and refrigerated at 4°C. The 5 d collected serum was packed in a cooler box at 15°C and transported to the University of Nairobi/KNH Paediatric laboratory and kept at a frozen state (below −20°C) until analysis to determine serum ferritin levels and C-reactive protein (CRP). Repeated cycles of freezing and thawing were avoided to retain the sample integrity.

### Biochemical methods for determining serum ferritin

‘Elegance Amplified Enzyme-Linked Immunosorbent Assay (ELISA)’ was used to quantitatively determine serum ferritin (SF). The determination was carried on LIASON^®^ Analyser (DiaSorin S.p.A. – Saluggia – Italy)^(13,14)^. CRP was analysed on HumaStar 600 machine (Wiesbaden, Germany) quantitatively by an immunoturbidimetric assay method. Human serum ferritin ELISA kits (Surgipath Services East Africa Ltd) and C-reactive protein ELISA kits (Chem Labs Ltd) and the standards were stored at a temperature of 2–8 °C. During analysis, the serum samples were retrieved from storage, defrost, and thoroughly mixed using a vortex mixer before running the tests. Lipemic or grossly haemolysed samples, harbouring alien materials such as cotton wool, clotted or those amounting to below 160 μL were altogether excluded from the analysis. However, none of the study's sample was eligible for exclusion. Participants were classified iron deficient (SF <15 μg/l or SF 15–70 μg/l and CRP >5 mg/l) otherwise normal ferritin levels^(32,33)^.

### Data analysis

Data on consumption patterns of iron- and ascorbate-rich foods and biomarkers were entered into SPSS software version 22 (Illinois, Chicago). Frequency and percentages were used to determine consumption patterns of iron- and ascorbate-rich foods. Multivariable regression was used to determine the association between consumption patterns of iron- and ascorbate-rich foods and iron status of WCA. A *P*-value of <0⋅05 was considered to be statistically significant. Confounding variables, such as parasitic infections and recent blood losses, were controlled for during analysis.

### Ethical considerations

The study was conducted according to the guidelines laid down in the Declaration of Helsinki and all procedures involving human participants were approved by the Kenyatta University Ethics Review Committee (PKU/2029/11176). Written informed consent was obtained from all respondents. Confidentiality and privacy of the respondents were ensured throughout the data collection and processing. A research permit was sought from the National Commission of Science Technology and Innovation (NACOSTI/P/19/2975).

## Results

### Consumption of iron-rich foods among the study respondents

Table 1 shows the consumption patterns for iron-rich foods among study respondents. Most of the iron-fortified food products were infrequently consumed (i.e. <2 times/week) by the respondents; fortified breakfast cereals (94⋅4 %), fortified brown chapatti (84⋅4 %), fortified porridge (57⋅5 %) and fortified refined *ugali* (53⋅8 %). Meat (61⋅3 %), sardines (61⋅9 %), chicken (80⋅0 %) and tilapia (82⋅5 %) were infrequently consumed. Among good sources of non-haeme iron frequently consumed included kale (76⋅3 %) and beans (50⋅6 %). However, most of the foods rich in non-haeme iron were infrequently consumed (i.e. <2 times/week); kunde and mrenda (cowpeas and jute mallow leaves) (79⋅4 %), kunde (77⋅5 %), terere (amaranth) (77⋅5 %), nderema (vine spinach) (76⋅3 %), pumpkin leaves (73⋅1 %), saget (spider plant) (68⋅8 %), green grams (66⋅9 %), spinach (66⋅9 %) and mrenda (63⋅1 %).

**Table 1.** Frequency of food consumption patterns of the study respondents

Characteristics	*n* 160	
	Infrequently (<2 times/week) (*n* (%))	Frequently (≥2 times/week) (*n* (%))
Cereals and their products		
Iron-fortified Porridge flour (mixture)	92 (57⋅5 %)	68 (42⋅5 %)
Unrefined *ugali* flour	77 (48⋅1 %)	83 (51⋅9 %)
Iron-fortified refined *ugali*	86 (53⋅8 %)	74 (46⋅3 %)
Iron-fortified brown *chapatti*	125 (84⋅4 %)	25 (15⋅6 %)
Iron-fortified breakfast cereals^a^	151 (94⋅4 %)	9 (5⋅6 %)
Meat and meat products		
Meat	98 (61⋅3 %)	62 (38⋅7 %)
Beef Samosa	145 (90⋅6 %)	15 (9⋅4 %)
Chicken	128 (80⋅0 %)	32 (20⋅0 %)
Sausages	138 (86⋅3 %)	22 (13⋅8 %)
Tilapia	132 (82⋅5 %)	28 (17⋅5 %)
Sardines	99 (61⋅9 %)	61 (38⋅1 %)
Legumes and Pulses		
Green grams	107 (66⋅9 %)	53 (33⋅1 %)
Beans	79 (49⋅4 %)	81 (50⋅6 %)
Green leafy vegetable		
*Terere* (amaranth)	124 (77⋅5 %)	36 (22⋅5 %)
Kale	38 (23⋅8 %)	122 (76⋅3 %)
*Mrenda* (jute mallow)	101 (63⋅1 %)	59 (36⋅9 %)
Pumpkin leaves	117 (73⋅1 %)	43 (26⋅9 %)
*Saget* (Spider plant)	110 (68⋅8 %)	50 (31⋅3 %)
*Nderema* (Vine Spinach)	122 (76⋅3 %)	38 (23⋅8 %)
Spinach	107 (66⋅9 %)	53 (33⋅1 %)
*Kunde* (Cowpeas leaves)	124 (77⋅5 %)	36 (22⋅5 %)
*Kunde* and *mrenda*^b^	127 (79⋅4 %)	33 (20⋅6 %)

a Iron-fortified breakfast cereals, i.e. cornflakes and Weetabix.

b *Kunde* and *mrenda* (Cowpeas and jute mallow leaves).

### Consumption of ascorbate-rich foods among WCA

Vegetables including spinach, kale, spider plant (Table 1) cabbages and capsicum (Table 2), and most fruits are considered to be a good source of ascorbate. The most frequently consumed fruits included bananas (48⋅1 %), oranges (45⋅6 %), mangoes (38⋅8 %) and avocados (31⋅3 %).

**Table 2.** Consumption of ascorbate-rich foods among the study respondents (fruits)

Characteristics	7-d Food Intake Frequencies (*n* 160)				Frequently
	Never *n* (%)	1 time/week *n* (%)	2–4 times/week *n* (%)	5–7 times/week *n* (%)	(≥2 times/week)
Fruits					
Oranges	41 (25⋅6 %)	46 (28⋅8 %)	53 (33⋅1 %)	20 (12⋅5 %)	73 (45⋅6 %)
Lemons	118 (73⋅8 %)	22 (13⋅8 %)	16 (10⋅0 %)	4 (2⋅5 %)	20 (12⋅5 %)
Orange juice	128 (80⋅0 %)	15 (9⋅4 %)	14 (8⋅8 %)	3 (1⋅9 %)	17 (10⋅6 %)
Pineapples	104 (65⋅0 %)	25 (9⋅4 %)	23 (14⋅4 %)	8 (5⋅0 %)	31 (19⋅4 %)
Guavas	125 (78⋅1 %)	23 (14⋅4 %)	9 (5⋅6 %)	3 (1⋅9 %)	12 (7⋅5 %)
Pawpaws	128 (80⋅0 %)	15 (9⋅4 %)	10 (6⋅3 %)	7 (4⋅4 %)	17 (10⋅6 %)
Apples	113 (70⋅6 %)	26 (16⋅3 %)	16 (10⋅0 %)	5 (3⋅1 %)	21 (13⋅1 %)
Mango	34 (21⋅3 %)	64 (40⋅0 %)	50 (31⋅3 %)	12 (7⋅5 %)	62 (38⋅8 %)
Avocado	77 (48⋅1 %)	33 (20⋅6 %)	24 (15⋅0 %)	26 (16⋅3 %)	50 (31⋅3 %)
Grapes	123 (76⋅9 %)	12 (7⋅5 %)	13 (8⋅1 %)	12 (7⋅5 %)	25 (15⋅6 %)
Banana	45 (28⋅1 %)	38 (23⋅8 %)	47 (29⋅4 %)	30 (18⋅8 %)	77 (48⋅1 %)
Watermelon	96 (60⋅0 %)	25 (15⋅6 %)	18 (11⋅3 %)	21 (13⋅1 %)	39 (24⋅4 %)
Vegetables					
Capsicum	126 (78⋅8 %)	12 (7⋅5 %)	16 (10⋅0 %)	6 (3⋅8 %)	22 (13⋅8 %)
Cucumber	121 (75⋅6 %)	9 (5⋅6 %)	22 (13⋅8 %)	8 (5⋅0 %)	30 (18⋅8 %)

### Deworming, malaria and recent blood loss status of the respondents

Over half of the study respondents (51⋅1 %) dewormed regularly (Table 3). Nearly all of the respondents (98⋅9 %) had not experienced an episode of malaria 2 weeks preceding the data collection. Among those who had major blood loss, 3⋅1 % of them experienced major blood loss within the previous 3 months before the study data collection.

**Table 3.** Deworming, malaria and recent blood loss status of the study respondents

Characteristics	*n* (%)
Frequency of deworming (*n* 94)	
Regularly (every 3 months)	48 (51⋅1 %)
Irregularly	46 (48⋅9 %)
Frequency of malaria episodes (*n* 160)	
Never	60 (37⋅5 %)
Before 2 weeks of data collection	97 (60⋅6 %)
Within 2 weeks preceding the data collection	3 (1⋅9 %)
Had recent (<3 months) major blood loss	5 (3⋅1 %)

### Serum ferritin levels of the respondents

After the adjustment of serum ferritin concentrations for inflammation (CRP > 5 mg/l), 45 % of the respondents were observed to be iron deficient (SF <15 μg/l or SF 15–70 μg/l and CRP > 5 mg/l) (Table 4).

**Table 4.** Iron status of study respondents

Iron status indicators	*n* 160 (*n* (%))
Inflammation marker	
C-reactive protein (CRP) levels (mg/l)	
Range of CRP levels (mg/l)	2⋅9–14⋅8
Mean [sd]	5⋅0 [2⋅1]
Elevated levels (CRP > 5 mg/l)	49 (30⋅6 %)
Serum Ferritin (SF) levels (μg/l)	
Range of SF levels (μg/l)	5⋅4–323
Mean (sd)	35⋅3 [42⋅2]
Crude Iron depleted stores (SF < 15 μg/l)	34 (21⋅3 %)
SF 15–70 μg/l and CRP> 5 mg/l	38 (23⋅8 %)
Adjusted^a^ iron stores status	
Iron deficient (ID) (SF <15 μg/l or SF 15–70 μg/l and CRP > 5 mg/l	72 (45⋅0 %)

a Adjusted iron stores for inflammation.

### Association between ascorbate-rich foods consumption and iron status among study respondents

To control for confounding variables, seven respondents were excluded from the logistic regression. Out of these seven, four had recent major blood loss and two had a frequent episode of malaria whereas one had both (Table 4). The association between consumption of ascorbate-rich foods and iron status is presented in Table 5. Respondents who consumed oranges 2–4 times/week had three times more chances of having normal iron status as compared to those who did not (AOR = 3⋅233, *P* = 0⋅009). Increasing the consumption of oranges to more than 5 times/week resulted in an elevated likelihood of fifteen (AOR = 15⋅283, *P* = 0⋅001) of having normal iron status. Consuming mangoes 2–4 times/week gave the respondents six times higher chances of normalising their iron status (AOR = 5⋅960, *P* < 0⋅001) whereas increasing the consumption to more than 5 times/week elevated the likelihood to nearly twenty-two (AOR = 21⋅750, *P* = 0⋅006).

**Table 5.** Association between consumption of ascorbate-rich foods and iron status^a^ among study participants

Determinants	*n* 153 AOR [CI]^b^	P*	Mean (sd)	CI (95 %)
Oranges				
Never (ref)		0⋅004	23.541 (15⋅051)	19⋅017–28⋅825
1 time/week	2⋅265 [0⋅906–5⋅659]	0⋅080	34⋅609 (61⋅643)	21⋅017–28⋅825
2–4 times/week	3⋅233 [1⋅338–7⋅809]	0⋅009	39⋅879 (35⋅606)	30⋅919–50⋅518
5–7 times/week	15⋅283 [3⋅029–77⋅115]	0⋅001	55⋅237 (37⋅385)	39⋅293–72⋅704
Pineapples				
Never (ref)		0⋅108	32⋅899 (28⋅513)	27⋅798–38⋅229
1 time/week	0⋅511 [0⋅208–1⋅254]	0⋅143	27⋅860 919⋅984)	20⋅400–35⋅545
2–4 times/week	1⋅629 [0⋅606–4⋅373]	0⋅333	58⋅945 (88⋅763)	30⋅709–104⋅792
5–7 times/week	5⋅019 [0⋅588–42⋅873]	0⋅140	41⋅775 (38⋅647)	21⋅971–70⋅477
Guavas				
Never (ref)		0⋅691	33⋅815 (31⋅006)	28⋅723–39⋅900
1 time/week	0⋅712 [0⋅061–8⋅271]	0⋅786	50⋅595 (85⋅333)	26⋅438–94⋅675
2–4 times/week	1⋅273 [0⋅096–16⋅971]	0⋅855	29⋅478 (22⋅746)	15⋅853–45⋅962
5–7 times/week	0⋅666 [0⋅043–10⋅393]	0⋅772	49⋅733 (32⋅458)	25⋅900–86⋅700
Mango				
Never (ref)		<0⋅001	31⋅837 (34⋅555)	21⋅423–47⋅059
1 time/week	1⋅662 [0⋅677–4⋅081]	0⋅268	25⋅358 (20⋅851)	20⋅753–31⋅228
2–4 times/week	5⋅960 [5⋅960–15⋅936]	<0⋅001	40⋅628 (25⋅354)	33⋅493–47⋅946
5–7 times/week	21⋅750 [2⋅457–192⋅525]	0⋅006	83⋅775 (116⋅531)	30⋅955–162⋅800

a The iron status indicator was serum ferritin levels.

b Adjusted for parasitic infections, major blood losses, and milk and milk products.

* Significance level at *P* < 0⋅05.

### Association between iron-rich foods consumption and iron status among the respondents

Respondents who used mixed porridge flour (millet, sorghum and/or cassava) once/week were four times (AOR = 4⋅249, *P* = 0⋅006) more likely to have normal iron stores whereas those that used it 2–4 times/week had nineteen times (AOR = 18⋅592, *P* < 0⋅001) higher likelihood (Table 6). Meat consumption was a statistically significant predictor of iron status demonstrated in the respondents who consumed meat once/week being three times more likely to have normal iron stores (AOR = 2⋅658, *P* = 0⋅016). However, the likelihood increased significantly to four among those who reported consuming meat 2–4 times/week (AOR = 4⋅450, *P* < 0⋅001). The consumption of sardines was demonstrated to significantly predict iron status. Women who subsisted on sardines once/week had seven times more chances (AOR = 7⋅365, *P* < 0⋅001) of having normal iron status whereas those who consumed it 2–4 times/week had nine times higher chances (AOR = 8⋅814, *P* < 0⋅001). Consuming beans once/week increased the likelihood of the women having normal iron status by four (AOR = 4⋅173, *P* = 0⋅007). The likelihood increased to nearly six times for those who consumed beans 2–4 times/week (AOR = 6⋅053, *P* < 0⋅001) and to thirteen times if the women consumed beans more than 5 times/week (AOR = 12⋅926, *P* = 0⋅005) (Table 6).

**Table 6.** Association between iron-rich foods consumption and iron status^a^ among study respondents

Determinants	*n* 153 AOR [CI]^b^	P*	Mean (sd)	CI (95 %)
Fortified Porridge flour (mixture)				
Never (ref)		<0⋅001	16⋅329 (11⋅363)	12⋅807–20⋅553
Once/week	4⋅249 [1⋅789–13⋅156]	0⋅006	24⋅019 (13⋅467)	20⋅935–28⋅191
2–4 times/week	18⋅592 [12⋅92–230⋅518]	<0⋅001	58⋅413 (66⋅887)	42⋅070–78⋅959
5–7 times/week	45⋅390 [8⋅216–250⋅772]	<0⋅001	50⋅855 (31⋅137)	37⋅879–64⋅546
Chicken				
Never (ref)		0⋅481	37⋅051 (52⋅947)	27⋅392–52⋅019
Once/week	0⋅595 [0⋅308–1⋅463]	0⋅179	34⋅325 (31⋅352)	26⋅280–43⋅139
2–4 times/week	0⋅835 [0⋅328–2⋅090]	0⋅693	38⋅407 (35⋅745)	26⋅998–52⋅588
5–7 times/week	0⋅289 [0⋅071–9⋅925]	0⋅324	32⋅567 (12⋅661)	19⋅600–44⋅900
Meat				
Never (ref)		<0⋅001	20⋅194 (22⋅532)	14⋅975–27⋅372
Once/week	2⋅658 [1⋅882–9⋅787]	0⋅016	30⋅597 (23⋅097)	24⋅997–36⋅926
2–4 times/week	4⋅410 [4⋅450–41⋅727]	<0⋅001	57⋅824 (64⋅329)	42⋅190–79⋅584
Sardines				
Never (ref)		<0⋅001	20⋅633 (21⋅343)	15⋅328–26⋅954
Once/week	7⋅365 [2⋅971–19⋅494]	<0⋅001	21⋅896 (9⋅965)	19⋅218–25⋅090
2–4 times/week	8⋅814 [6⋅690–63⋅511]	<0⋅001	43⋅809 (24⋅948)	37⋅514–52⋅038
Beans				
Never (ref)		0⋅002	21⋅597 (17⋅123)	15⋅633–27⋅957
Once/week	4⋅173 [1⋅514–10⋅843]	0⋅007	32⋅971 (31⋅352)	25⋅077–42⋅234
2–4 times/week	6⋅053 [2⋅850–20⋅028]	<0⋅001	42⋅130 (54⋅982)	31⋅272–57⋅996
5–7 times/week	12⋅926 [2⋅192–182⋅442]	0⋅005	56⋅210 (40⋅651)	34⋅712–81⋅899

a The iron status indicator was serum ferritin levels.

b Adjusted for parasitic infections, major blood losses, and milk and milk products.

* Significance level at *P* < 0⋅05.

The association between the consumption of dark green leafy vegetables and iron status of respondents is presented in Table 7. Respondents who consumed amaranth once/week had 2⋅5 times higher chances (AOR = 2⋅534 *P* = 0⋅019) of having normal iron stores whereas those who consumed it 2–4 times/week had seven times higher chances (AOR = 7⋅141, *P* = 0⋅001). The likelihood increased to nine times if the respondents consumed amaranth more than 5 times/week (AOR = 8⋅818, *P* = 0⋅008). Consuming spider plants once/week gave the respondents three times higher chances (AOR = 3⋅290, *P* = 0⋅007) of developing normal iron stores as compared to those who never took the vegetable. Increasing the intake of spider plants to 2–4 times/week elevated the likelihood to twenty-two of having normal iron stores (AOR = 22⋅132, *P* < 0⋅001) (Table 7).

**Table 7.** Association between dark green leafy vegetable consumption and iron status^a^ among study respondents

Determinants	*n* 153 AOR [CI]^b^	P*	Mean (sd)	CI (95 %)
Amaranth				
Never (ref)		0⋅001	34⋅199 (54⋅241)	23⋅891–49⋅283
Once/week	2⋅534 [1⋅166–5⋅507]	0⋅019	28⋅102 (24⋅498)	22⋅257–36⋅212
2–4 times/week	7⋅141 [2⋅153–23⋅688]	0⋅001	41⋅930 (24⋅763)	32⋅586–52⋅349
5–7 times/week	8⋅818 [1⋅766–42⋅044]	0⋅008	67⋅585 (40⋅871)	46⋅028–90⋅643
Kale				
Never (ref)		0⋅348	21⋅980 (10⋅326)	15⋅835–27⋅819
Once/week	2⋅888 [0⋅606–13⋅762]	0⋅183	51⋅515 (82⋅9161)	27⋅192–88⋅016
2–4 times/week	3⋅691 [0⋅878–15⋅524]	0⋅075	33⋅813 (29⋅032)	27⋅608–40⋅625
5–7 times/week	2⋅835 [0⋅637–12⋅613]	0⋅171	34⋅277 (26⋅278)	26⋅779–43⋅110
Jute mallow				
Never (ref)		0⋅832	26⋅918 (22⋅384)	21⋅979–33⋅649
Once/week	1⋅138 [0⋅498–2⋅599]	0⋅760	47⋅364 (70⋅938)	29⋅520–72⋅947
2–4 times/week	1⋅636 [0⋅710–3⋅767]	0⋅247	39⋅328 (32⋅379)	30⋅902–49⋅217
5–7 times/week	2⋅290 [0⋅557–9⋅415]	0⋅251	32⋅325 (15⋅115)	24⋅076–41⋅358
Spider plant				
Never (ref)		<0⋅001	25⋅840 (25⋅738)	19⋅115–34⋅417
Once/week	3⋅290 [1⋅395–7⋅756]	0⋅007	26⋅792 (17⋅009)	22⋅713–31⋅173
2–4 times/week	22⋅132 [6⋅831–71⋅708]	<0⋅001	57⋅715 (70⋅575)	39⋅105–83⋅983
5–7 times/week	21⋅855 [2⋅379–200⋅815]	0⋅006	54⋅988 (19⋅123)	41⋅300–68⋅400
Spinach				
Never (ref)		0⋅382	41⋅530 (56⋅215)	30⋅723–55⋅544
Once/week	0⋅521 [0⋅219–1⋅241]	0⋅141	35⋅974 (29⋅676)	25⋅789–47⋅676
2–4 times/week	0⋅699 [0⋅301–1⋅623]	0⋅405	29⋅600 (26⋅757)	22⋅314–38⋅846
5–7 times/week	1⋅297 [0⋅877–1⋅917]	0⋅964	28⋅940 (17⋅233)	21⋅281–38⋅833

a Adjusted for parasitic infections, major blood losses, and milk and milk products.

b The iron status indicator was serum ferritin levels.

* Significance level at *P* < 0⋅05.

## Discussion

The study found that iron deficiency prevalence was at 45 % among the study respondents. The observed prevalence is more than double of the national iron deficiency prevalence reported to be 21⋅3 % in 2011^(1)^. The high prevalence in the study area suggests the prevailing of unique determinants that explain the phenomena. Many factors determine the iron status of WCA, however, the present study investigated the consumption patterns of iron- and ascorbate-rich foods as a determinant.

The study found that an increase in the consumption of meat, sardines, fortified mixed porridge, beans, amaranth and spider plants significantly increased the likelihood of respondents having normal iron status. Meat and sardines^(24)^ are a rich source of haeme iron. A big advantage posed by taking haeme iron is that it is highly bioavailable^(34)^ and enhances the absorption of non-haeme iron^(35)^. Several studies have reported a significant dependency of iron status on haeme iron among women^(36,37)^ or meat intake^(36,38–40)^. Contrary, some studies found no association between meat intake and iron status among young women^(23,41)^. However, most of the respondents infrequently consumed meat probably due to its high cost. Similarly, Kenyan women have been noted to consume chicken and meat less frequently^(13,16,17)^. A study conducted in Mumbai among WCA found that the average intake of meat and their products were low^(18)^. The fact that animal products were generally unpopular among women could compound efforts of supplying sufficient iron to the women's body as animal products provide haeme iron that has higher bioavailability as compared to non-haeme iron^(20)^.

Dark green leafy vegetables are a good source of non-haeme iron^(24)^. Only kale was frequently consumed vegetable. A Kenyan study reported that vegetables were poorly consumed with women mostly subsisting on kale, which they ate 4 times/week^(14)^. Similarly, poor consumption of vegetables has been reported in Bangladesh^(19)^ and in third-world countries^(15)^. A high intake of beans was observed among the respondents, which is in line with the finding of previous Kenyan studies among women^(13,16)^. Plant-based food products are noted to supply non-haeme iron that, unfortunately, is not readily available for absorption especially when bioactive phenolic compounds such as tannins are present^(20)^. However, the absorption of non-haeme iron can be enhanced by not only the endogenous ferrireductase but also by exogenous reducing agents such as ascorbic acid^(42)^.

Ascorbic acid is a powerful enhancer of iron absorption^(42,43)^. Its mechanism is well-understood as it converts ferric iron to ferrous iron in the gastrointestinal tract to constitute soluble complexes at the low pH of the gastric section to enable iron absorption^(22)^. Vegetables and fruits are rich sources of natural ascorbic acid^(23)^ and, therefore, their consumption is very significant in enhancing iron absorption. The present study found out that an increase in the consumption of oranges and mangoes gave the respondents a correspondingly high likelihood of having normal iron status. These two fruits together with bananas were the most consumed by the respondents. Their consumption is encouraged because they are readily available in the Kenyan market alongside apples, pawpaws, pineapples and avocados^(25–27)^. Therefore, the consumption of oranges, mangoes, avocados and apples was more likely to be affected by price fluctuations rather than by seasonality.

However, infrequent consumption was registered among orange juices, pineapples, guavas, apples, watermelon, grapes and avocados. Many factors could influence the consumption of fruits including seasonality of their production^(26)^, distribution channels^(29)^, and whether they are local or exotic as well as the price fluctuations. Similarly, Kenyan studies have unfortunately reported low consumption of fruits among women^(16,27)^. An Indian study also reported that rural women eat fruits infrequently and below the recommended level^(29)^. The fact that most of the respondents infrequently consumed these fruits could explain why they did not predict their iron status.

Studies have reported a positive association between dietary ascorbic acid^(36,44)^, fruits^(36,45,46)^ and fruit juices^(45)^ intake and iron status among WCA. However, the majority of the previous studies have reported no association between iron status among young women and overall intake of ascorbic acid^(23,37,38,47,48)^, intake of fruits^(23,37,47,48)^ or fruit juices^(23,42)^. Possible explanations for no association between ascorbate-rich foods and iron status reported by most studies may include poor preparation practices and timing of fruits, method of cooking vegetables and frequency of consumption.

The study has limitations in that it did not assess the nutritional knowledge on the consumption of iron- and ascorbate-rich foods among the respondents. Nutritional knowledge may confound the relationship between dietary patterns and iron status among the respondents. Another limitation of the study is that it did not determine chronic inflammation among the respondents to allow for correction of iron status based on levels of serum ferritin. Also, the study included age 15 women who are under adolescence and predisposed to suffer from the triple burden of iron-deficient anaemia.

## Conclusion

The present study investigated the relationship between consumption patterns of iron- and ascorbate-rich foods and iron status among WCA. Four to five in every ten WCA in Nandi County were iron depleted. The respondents infrequently consumed iron- and ascorbate-rich foods. Consumption patterns of iron-rich foods (iron-fortified porridge, meat, sardines, beans, amaranth and spider plants) positively predicted the normal iron status of WCA. Ascorbate-rich foods (oranges and mangoes) consumption positively predicted the iron status of WCA. For better iron status outcome, WCA and other risk groups should consume at least 2 times/week of each food rich in iron and ascorbate, i.e. meat and meat products, fortified food products, citrus fruits and dark green vegetables.
